# Supplementary material for: surveydown: An open-source, markdown-based platform for programmable and reproducible surveys
Source: PLoS One. 2025 Aug 29;20(8):e0331002. doi: 10.1371/journal.pone.0331002 (PMC12396762; doi:10.1371/journal.pone.0331002)
Supplement: Attachment — Submitted filename: r2r.pdf [file pone.0331002.s001.pdf]

Dear reviewers,

We greatly appreciate your comments and suggestions on our manuscript. We have revised our manuscript that takes these suggestions into consideration, and we also provide a point-by-point response to your suggestions below. Thank you again for your time in reviewing our manuscript, and we look forward to seeing your comments on these revisions.

Regards,
Pingfan Hu and John Paul Helveston

Reviewer #1:

The authors present an R package for building and managing surveys using a markdown based system. The distinguishing features of the package over existing methods is the comprehensive nature of the package, including git version control and real-time code execution through shiny and control over the implementation details without needing web development skills. The package generates the web application completely using R which is very useful for data scientists who are often unfamiliar with writing html and using javascript frameworks.

> We appreciate the acknowledgement of what our surveydown package offers.

I found the design to be very well thought out and logical, using existing formats and well established standard packages Quarto, Shiny and Postgres. There is a useful and extensive discussion of the existing packages and the differences which was clear and compelling, especially with the differences over the most obvious alternative - Google Forms. I found that the focus on making it reproducible through being able to specify everything through code is extremely useful for those trying to tie surveys with other software packages. The authors also state that the package could have a GUI front-end if necessary which also enhances its utility. The availability of templates for surveys would also encourage adoption.

> Yes, we are already working on a GUI application, which is an additional R package we are calling sdstudio (https://github.com/surveydown-dev/sdstudio). It is actually another shiny app that is used to support a no-code solution for creating and managing surveydown surveys. It also supports live-preview and data management. We added a little more detail in our revised manuscript on this effort since we have made more progress on it since our original submission, writing on the second to last paragraph in the manuscript the following:
>
> “We are in the process of building this tool as a companion package called sdstudio (https://github.com/surveydown-dev/sdstudio), which will serve as a comprehensive studio for building, previewing, and managing surveys using a GUI while maintaining full reproducibility.”

Reviewer #2:

I think surveydown can be a great tool for easy survey programming. I just have a few outstanding issues about the software and about the manuscript.

Concerning your app.R file: suppose that I make multiple surveys with surveymarkdown and run sd_ui(). How does R know which .qmd file I want to run? In other words, what connects a particular app.R document to a particular survey.qmd document? Is it just that they need to be in the same directory/project? And if so, can each directory only accommodate one survey.qmd document? (I would not perceive this as a shortcoming of the software, as one can always just make more directories; I'd just like it spelled out for readers.)

> That's a really good question, and we should have clarified that in our original manuscript. The intended usage is to have the app.R and survey.qmd files both in a single folder to define one survey project. Each survey should be in its own folder, so if a user wishes to make multiple surveys, they should keep them organized into folders. This structure allows the package to "know" which files to use for each survey. This was a design choice we made to avoid unintended actions, such as sd_ui() rendering the wrong file, etc. We clarified this in our revised submission after Figure 1:
>
> "Every surveydown survey consists of a survey document and a web application, defined in two separate files named **survey.qmd** and **app.R**. These files must be in the same directory and have these precise file names as the surveydown package searches for them in the working directory. To make multiple surveys, users should organize each survey into a separate folder."

An issue that should not be left to be figured out later is data security. Platforms like Qualtrics use TLS encryption for data transmission. You envision that surveydown platforms will deploy on using either personal or third-party hosting services, who then pass responses from a deployed Shiny app back to the survey creator's PostgreSQL database. Does the encryption and security with which that information is passed vary by hosting provider? If so, can you shed some light on which hosting platforms offer that kind of encryption, and/or which ones may not be appropriate for surveys that aim to collect sensitive information?

I see that you explicitly recommend supabase for SQL storage. Can you clarify the security features on supabase?

> This is a good observation. While we have some documentation on security on our main documentation site

(https://surveydown.org/docs/security), we should have added more in our manuscript to explain this as it is very important. Given the disaggregated design of surveydown, security has several points to consider: the surveydown application code, the shiny app hosting service, and the data storage service. We detail each in the section we added to the revised manuscript, which we added at the end of section 3, copied below for convenience here.

"Finally, it is important to note security considerations for data collection tools like surveydown. Given surveydown's disaggregated design, three separate components require security considerations: the surveydown application code, the app hosting service, and the data storage service. For the surveydown application code, we have followed best practices in how survey response data is internally handled, such as using SQL injection prevention strategies and ensuring that users store their database credentials as a .env file to avoid accidental exposure. We also adopted an architecture where all content in the survey is served entirely from the shiny server, preventing respondents from being able to see content in the source code of other pages before getting there from the survey navigation. While the package does not yet have a security compliance certificate for the application code, this is a longer-term goal. For the app hosting service, users can choose from different providers, each of which offer different security measures. For example, while shinyapps.io is a free service, it is not HIPPA compliant. Alternatives such as Heroku or Hugging Face may offer other security measures, and users are encouraged to review their security needs before choosing a hosting service. Finally, for data storage, we suggest Supabase as a free, open-source, and convenient to use platform that has TLS encryption among other security features, including multi-factor authentication (MFA) and being SOC 2 and HIPAA compliant."

Make sure to update the GitHub figures (stars, issues, discussions) for the next iteration of the manuscript.

Thanks, we have done this.

The section on reproducibility of code doesn't give enough credit to the inter-interpretability of other survey platforms. My survey platform of choice is typically Qualtrics, and other researchers who use Qualtrics can pretty easily figure out the structure of eachother's Qualtrics surveys by reuploading .qsf files into Qualtrics and reading the survey structure in the GUI. This section could be improved by just clarifying that with plain text structuring, this sort of inter-interpretability arises *even without access to proprietary software* (like Qualtrics).

This is a good suggestion. We took your advice and added the following:

"Alternative survey platforms do support different forms of reproducibility, but often in limited ways. For example, users of the proprietary software Qualtrics [4] export a **.qsf** file to share survey designs with other Qualtrics users to reproduce their surveys. However, this only enables reproducibility for users who have a Qualtrics subscription, which limits accessibility and interoperability with version control tools like Git. In contrast, by using plain text files, surveydown surveys can be easily read and directly edited without the need for proprietary software to interpret the files."

In Section 3.3, you talk about using conditional logic via Shiny. It's clear that a page can be displayed only if a logical condition is true, but how could a user reverse the logical condition being true to view older pages and change answers? In other words, how do you implement a 'back button' in surveydown? This is a feature many social scientists consider necessary in their surveys, especially if they arise from complex experiments.

At the moment, we have not yet implemented support for a "back" button, precisely for this reason. If a designer applies a conditional skip forward logic, for example, then pressing a back button to return to a prior page would require considering the skip logic that led to the current position in the survey. It can be easy to get stuck in a navigational loop this way. This is on our roadmap though as we agree this may be a necessary feature for some settings. We added the following in the revised manuscript just before Figure 2 to clarify that backward navigation is not yet supported:

"For now, surveydown only supports forward navigation as backwards navigation requires careful consideration of potential skipping logic that can create navigational loops, though adding support for a back button is on the development roadmap."

Relatedly, if a 'back button' is possible in surveydown, how do survey designers control logical conditions that hold randomly? Survey platforms like Qualtrics will fix a random seed for each individual participant, preventing someone from changing their randomly-assigned treatment status by using the back button and receiving another random draw. How can survey users implement similar protocols in surveydown?

Something similar can be achieved with surveydown. For example, we have a section on our main documentation page on randomization that shows several approaches for randomizing what each individual respondent sees: https://surveydown.org/docs/randomization

In short, each respondent sees a unique session, and a unique value can be assigned to each session such that their version can be “fixed” for that user or continuously randomly re-created - it’s up to the survey designer’s choice. We also use browser cookies to restore the session state so that users will see the same version even if they refresh the page or lose connectivity. We added a small paragraph noting this just before Figure 4:

“Finally, the Shiny framework enables a wide variety of randomization options in how it handles sessions. Respondents can be assigned random values that are held constant for each user or not depending on the survey designer's objective, providing a high degree of flexibility in randomized survey designs.”

Prose error on line 292: “We call these questions ‘reactive question’”

Thanks, we corrected this typo. Our current version now reads:

“We call these ‘reactive questions,’ which are defined in the **app.R** file and called in the **survey.qmd** file using sd_output().”

I think the live-polling feature of surveydown could make it a useful replacement for proprietary live-polling platforms such as Menti. Do you agree? If so, I think this is a use case worth highlighting, as universities currently shell out considerable funds to give teachers collective access to such GUI-based software.

Yes! Live polling is yet another unique use case that surveydown can certainly implement. More generally, with surveydown users can access all of the response data in real-time during the survey, so live polling is really just a subset of this more general feature. Taking your advice, we highlighted this feature in the revised manuscript near the end of the Comparison section writing:

“Given the flexibility of the Shiny web framework, surveydown can also serve as a free and open-source alternative to existing proprietary platforms for more specialized purposes. For example, the Poll Maker by Mentimeter [15] is a popular proprietary platform for creating interactive live polls and quizzes, where respondents see the live survey results in real time. A similar live polling capability can be achieved with surveydown using the sd_get_data() function, which gets the latest response data and refreshes according to a specified time interval, which can then be used to display summary results to respondents. A live-polling template is available at https://surveydown.org/templates/live_polling.”

Table 1 is useful but the categorization is rather vague. Rather than binning platforms into each category via ‘yes’, ‘partially’, and ‘no’ labels, the paper would benefit from these labels being expanded out, explaining what you mean in each. This also gives users a clearer demonstration of the differences between software they’re already using and a software they might want to switch to (say, surveydown).

> After discussion, we chose to keep the table in place, though we improved the symbols used to make the table easier to read. We felt the table was helpful for making a quick comparison, as including a more detailed description in each table cell would make the table more difficult to read. However, we added a more thorough description of what we meant by each categorization in the paragraph after the bullet list in section 3.4, which reads:
>
> “Table 1 compares 14 platforms across these dimensions. For the last four features, we label the feature as "Yes", "No", or "Partially", in which case "Partially" means the platform has some limited capabilities for the feature. For **Reproducibility**, we label a platform as "Partial" if surveys cannot be freely reproduced without proprietary software. For example, while Qualtrics surveys can be reproduced using .qsf files, only Qualtrics subscribers can use them. For **Data Control**, we label a service as "No" if users can only obtain access to the response data through a proprietary service, and "Partial" if the service offers the capability of storing the data on a private server, which might require a customized or more complex set of steps compared to the service storing the data.”

I don’t think it’s fair to single out LimeSurvey for requiring users to know MySQL for customization when your software requires PostgreSQL for data management.

> This is a fair critique. We revised our text accordingly:
>
> “Notable open-source alternatives to surveydown include formr [10], which also integrates with R but requires a complex server setup for self-hosting; LimeSurvey [12], which offers extensive features as a GUI-based platform, and Open Data Kit [13], which excels in field-based mobile data collection but creates a disconnect between survey design and analysis environments.”

In the conclusion, what do you mean by ‘built-in panel management’ and ‘advanced quota management’?

> We revised this to clarify, it now reads “From a functionality perspective, although surveydown can currently handle complex survey designs, it

does not yet match all specialized features of mature platforms, especially with respect to participant recruitment and tracking as some platforms do (e.g. Qualtrics).”
