# Supplementary material for: surveydown: An open-source, markdown-based platform for programmable and reproducible surveys
Source: PLoS One. 2025 Aug 29;20(8):e0331002. doi: 10.1371/journal.pone.0331002 (PMC12396762; doi:10.1371/journal.pone.0331002)
Supplement: Attachment — Submitted filename: r2r_auresp_2.pdf [file pone.0331002.s002.pdf]

Dear reviewers,

We have revised the manuscript considering your suggestions. Please see our response below. We appreciate your time in reviewing our manuscript as well as your suggestions!

Regards,
Pingfan Hu and John Paul Helveston

# Reviewer #1:

I found the revisions to be useful and the comments helpful in understanding the scope and rationale for their software.

We appreciate your reviews that helped build up the quality of our paper.

# Reviewer #2:

This looks good to go on two conditions. (1) Parse for spelling in the new additions (e.g., HIPPA vs. HIPAA). (2) expand your definition of 'programmable' in Table 1 to include a 'partial' category to include lightly-programmable software like shinysurveys, and provide a good definition of partial programmability for Table 1.

1) Yes, the correct spelling for this term is HIPAA (Health Insurance Portability and Accountability Act). After careful spell check, we've also located the misspellings of other words. They are colored in green as you can see in our "marked" version.

2) Yes, now we've marked shinysurveys as partially programmable in the table. We modified our definition of "programmable" in the bullet list in section 3.4 as "The ability to embed and execute custom code during survey runtime, enabling programmatic control over content display, data processing, and user interactions." In the paragraph describing shinysurveys, we added the following: "A close alternative to surveydown is shinysurveys package, by Trattner and D'Agostino McGowan (2021), which offers a more formalized implementation comparable to Google Forms, **and light programmability support with R code**." We also expanded the definition for "Programmable": The ability to create dynamic content by having custom code run during the survey, **or to inject code scripts reinforcing the survey content creation**.
